# β-catenin, PAX2, and PTEN Aberrancy Across the Spectrum of Endometrioid Ovarian Lesions

**DOI:** 10.1097/PGP.0000000000001046

**Published:** 2024-07-31

**Authors:** Maria M. Del Mundo, Mitzi Aguilar, Hao Chen, Shuang Niu, Subhransu S. Sahoo, Sambit Roy, Wenxin Zheng, Elena Lucas, Diego H. Castrillon

**Affiliations:** * School of Medicine, UT Southwestern Medical Center, Dallas, TX.; † Department of Pathology, UT Southwestern Medical Center, Dallas, TX.; ‡ Department of Obstetrics and Gynecology, UT Southwestern Medical Center, Dallas, TX.; § Harold C. Simmons Comprehensive Cancer Center, UT Southwestern Medical Center, Dallas, TX.

**Keywords:** Ovarian cancer, Endometriosis, Atypical endometriosis, Endometrioid borderline tumor, Endometrioid adenocarcinoma of the ovary, β-catenin, PAX2, PTEN

## Abstract

Endometriosis is a common condition, with the ovary being the most common anatomic site. Endometriosis—particularly in the ovary—is associated with a risk of malignant progression, with a histologic spectrum of lesions from benign to malignant. Recently, a panel of 3 markers consisting of β-catenin, PAX2, and PTEN has been described as a potentially useful diagnostic adjunct in the diagnosis of intrauterine endometrioid neoplasia, where aberrancy for one or more of the markers is strongly associated with neoplasia. Here, we applied the panel to ovarian endometrioid lesions, including endometriosis, endometriosis with flat cytologic atypia, endometrioid borderline tumors, and endometrioid adenocarcinoma (n = 85 cases in total). The incidence of aberrancy for the 3 markers increased along this putative neoplastic spectrum, arguing for a role of each of the markers in the neoplastic transformation of ovarian endometriosis. Just 1/32 (3%) of cases of nonatypical endometriosis was marker-aberrant, and this case was aberrant only for PAX2. One of 5 cases (20%) of endometriosis with atypia was marker-aberrant (both PAX2 and PTEN), supporting prior findings that some cases of flat atypia may represent bona fide precursor lesions. Of 19 endometrioid borderline tumors, 10 (53%) were aberrant for one or more markers, with PAX2 being the most frequently aberrant. Of 29 endometrioid adenocarcinomas, 28 (96.6%) were aberrant for at least 1 marker, with PAX2 again the most frequently aberrant. Patterns of aberrancy were well-preserved in areas of nonatypical endometriosis adjacent to borderline tumor or adenocarcinoma, supporting a biological origin in a common marker-aberrant precursor. The findings show that the biomarker panel could be of some diagnostic utility in the characterization of ovarian endometrioid neoplasia, such as in the diagnosis of endometrioid borderline tumor, distinguishing endometrioid from nonendometrioid lesions, or in identifying other types of early precursors at a higher risk of malignant transformation.

Endometriosis is a common condition affecting 10% of reproductive-age women and is believed to result from the retrograde transit of menstrual endometrial fragments through the fallopian tube and fimbrial opening into the peritoneal cavity^1,2^. Consistent with its anatomic location and physical contact with fimbria, the ovary is the most common site of endometriosis, although the disease is frequently multifocal and involves other abdominopelvic sites. Endometriosis can undergo malignant transformation, particularly in the ovary, likely due to its association with inflammation and other poorly understood factors^3^. An estimated 0.5% to 1% of cases are complicated by neoplasia^4^.

A spectrum of endometriosis-associated lesions ranging from potentially to definitively neoplastic has been described. Some cases of endometriosis/endometriomas have flat epithelium with nuclear atypia. Mild atypia is common and likely reactive, but the atypia is on occasion severe and striking, raising concerns for neoplasia. Other endometriosis-associated lesions exhibit gland crowding and related architectural abnormalities characteristic of endometrioid intraepithelial neoplasia/atypical hyperplasia (EIN/AH). The term “atypical endometriosis” has been used for both of these types of lesions even though they likely differ in biological behavior and clinical significance^5,6^, leading some investigators to argue against the use of the term^7^. Here, we use “endometriosis with atypia” (EA) to refer to those lesions with flat cytologic atypia alone and “endometrioid borderline tumor” (EBT) to refer to lesions with architectural abnormalities resembling EIN/AH. Both EA and EBT can occur in association with adenocarcinoma and may harbor genetic driver mutations^8^. EBT has more generally been considered a definitive neoplasm, while EA remains less defined and its diagnostic significance more controversial^5,6,8^. Mutations in the *PTEN* gene are frequent in EBT, consistent with the idea that this is an early driver event in ovarian endometrioid neoplasia^9^, as is the case in endometrium^10–12^. *CTNNB1* (encoding β-catenin) mutations have also been documented in EBT^13^. For EA, some studies have found no risk of future malignancy, while others have reported such an association^5^. Surgical removal of an endometriotic cyst or the entire ovary could eliminate the risk of malignant progression, however, complicating efforts to assess the significance of EA as a potential precursor lesion.

Building on earlier studies^11,14–17^, we recently defined a panel of 3 immunohistochemical markers—comprised of β-catenin, PAX2, and PTEN—with practical utility in the diagnosis of EIN/AH^18,19^. Reflecting their unique biology, each marker is scored per distinct criteria^20–22^. β-catenin, PAX2, and PTEN are each aberrant in more than half of EIN/AH; with a panel of all 3, at least 1 is aberrant in 93% of cases^18,23–25^.

Here, we sought to explore patterns of aberrancy for the 3 biomarkers across a spectrum of ovarian endometrioid lesions, with the goal of exploring the utility of the biomarker panel in endometrioid lesions of the ovary, as well as gaining potential insights into their status as neoplastic precursor lesions.

## MATERIALS AND METHODS

### Case Selection

After approval from the UT Southwestern IRB, cases at the UT Southwestern Clements University and Parkland Hospitals were retrospectively identified through text searches. EBT was defined per WHO 2020 criteria: closely packed, crowded endometrioid glands, but falling short of the criteria for adenocarcinoma^26^. EA was defined as the presence of high-grade nuclear atypia visible at low magnification in endometrioma/endometriosis without overt architectural features of neoplasia (ie, gland crowding). Characteristic nuclear features included pleomorphism, angular shapes, hyperchromasia, and smudging. For EBT, seromucinous differentiation was allowed (ie, not an exclusion criterion), and 14 of the 19 cases of EBT exhibited features of seromucinous borderline tumors. Endometrioid adenocarcinomas were defined per standard histologic criteria, including back-to-back glands. The most representative tissue block for each case was selected for immunostaining. In 3 cases where analysis of a potential benign precursor (ie, endometriosis) required IHC of another block, IHC was performed on the additional block.

### Immunohistochemistry (IHC)

PAX2, PTEN, and β-catenin staining protocols previously validated for clinical testing were performed on 4 μm sections in the clinical immunohistochemistry laboratory on a DAKO Autostainer Link 48 instrument. The following primary antibodies were used: β-catenin (prediluted, clone β-catenin-1, #IR70261–2, Agilent), PAX2 (prediluted, clone EP235, #BSB2567, Cancer Diagnostics, Durham, NC), and PTEN (prediluted, clone 6H2.1, #PM278AA, BioCare, Pacheco, CA) with antigen retrieval performed in low pH (6.0) for β-catenin and high pH (9.0) Tris/EDTA solution (Agilent) for the other markers at 97°C for 20 minutes. FLEX peroxidase block was performed for 10 minutes for β-catenin and 5 minutes for other markers. Primary antibody incubation time was 20 minutes for β-catenin, and 40 minutes for PAX2 and PTEN. Incubation with Mouse Linker (Agilent) for β-catenin and Rabbit Linker (Agilent) for PAX2 was performed for 10 minutes. Secondary antibody (Envision/HRP) incubation time was 20 minutes for PTEN, β-catenin, and 30 minutes for PAX2. For all antibodies, the enzymatic conversion of the 3,3′-diaminobenzidine tetra-hydrochloride chromogen was performed for 10 minutes at room temperature.

### Scoring Criteria for the 3 Markers by IHC

#### β-catenin

Aberrancy is manifested as nuclear localization versus its normal membranous/cytoplasmic localization, often associated with overexpression. Strong nuclear β-catenin is scored as aberrant, even if focal^20,21^. Nuclear staining is assessed only in glandular epithelium (not “squamous” morules), since true morules always exhibit nuclear β-catenin^27^. Low levels of nuclear β-catenin are normal; the criterion for strong nuclear expression is nuclear staining clearly greater than that of the lateral cell membranes^18^.

#### PAX2

PAX2 is a nuclear transcription factor expressed within the endometrial epithelium, and scoring aberrancy requires complete loss of expression within all the nuclei in an entire gland in a cross-section. Decreased expression is not scored as aberrant. In most specimens, residual normal glands that retain PAX2 serve as internal controls for PAX2 IHC.

#### PTEN

PTEN is ubiquitously expressed in endometrial glands, stroma, and leukocytes, and true loss within the endometrial glandular epithelium gives rise to a “punched-out” appearance of glands relative to the surrounding stroma. Leukocytes interspersed within endometrial glands, which can be abundant, retain PTEN expression even when true epithelial loss is present.

Focal loss of PAX2 and PTEN in individual glands or small clusters of glands can occur in normal endometria, with > 10% loss across the glands in the entire specimen used as the cutoff for aberrancy. However, most cases of EIN/AH exhibit PAX2 or PTEN loss in > 25% of glands, making scoring more straightforward.

In endometriotic cysts, the criterion for loss for PAX2 or PTEN was loss across > 50% of the cyst epithelium.

## RESULTS

PAX2, PTEN, and β-catenin immunostains were performed on endometriosis-associated endometrioid ovarian lesions from a total of 85 patients, including 32 usual-type (nonatypical) endometriosis/endometriomas, n = 5 EA, n = 19 EBT, and n = 29 endometrioid adenocarcinomas (n = 16 FIGO Grade 1, n = 10 Grade 2, and n = 3 Grade 3). Patient demographics are summarized in Table 1. Scoring for each immunostain was performed per published criteria (see Methods)^12,19,23^.

**FIGURE 1.**
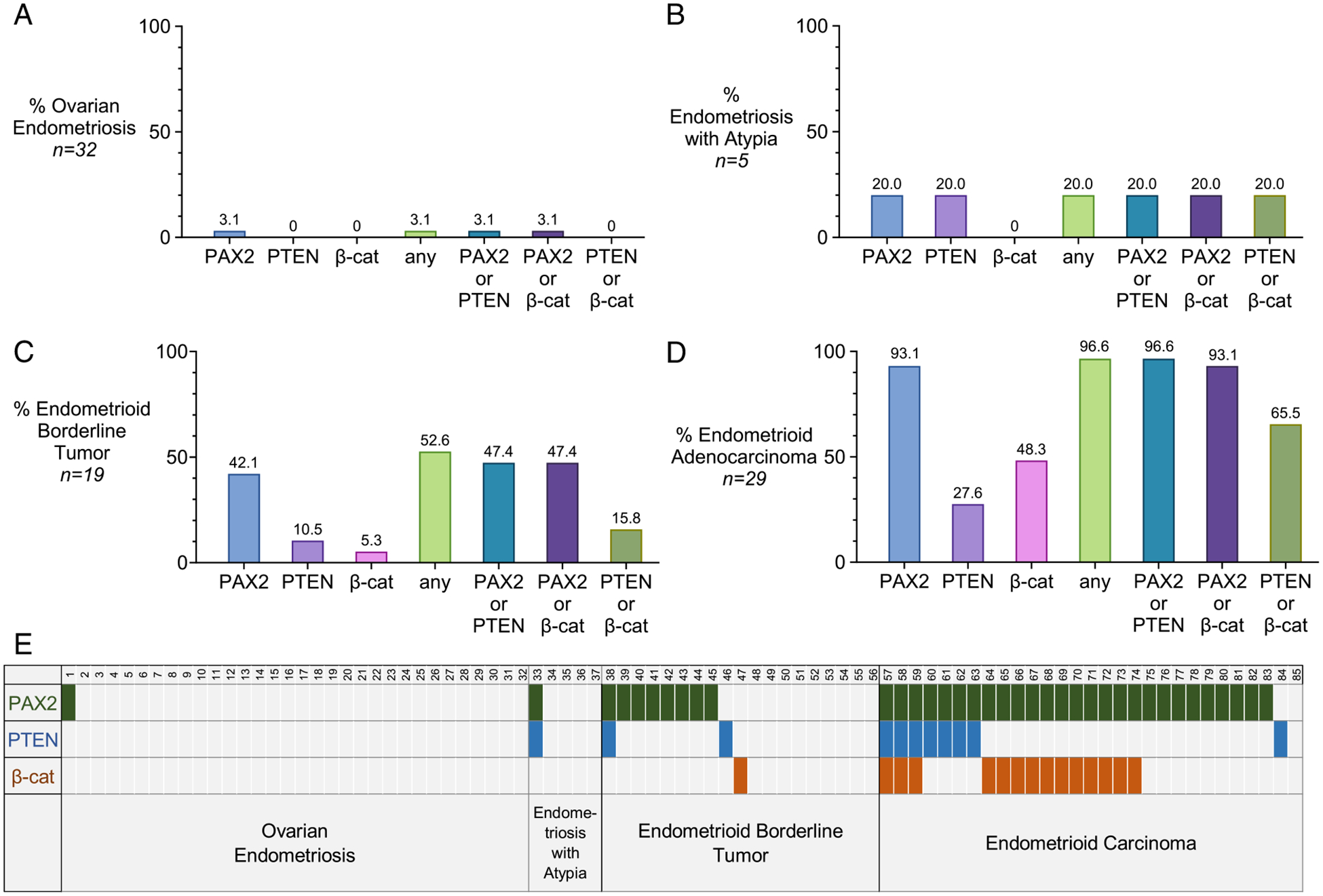
Marker aberrance across diagnostic categories. (A-D) Bar graphs for the 4 diagnostic entities. Percentages appear above each bar. (E) Heat map showing patterns of marker aberrancy in each of the 85 cases.

The results are shown in Fig. 1 for the 4 diagnostic categories. Marker aberrance was rare in ovarian endometriosis without atypia, limited to a single case (1/32) aberrant for only PAX2 (Fig. 1A). Marker aberrance occurred in 20% of EA, with 1 case being aberrant for both PAX2 and PTEN (Fig. 1B). Marker aberrance was more frequent in EBT, with PAX2 having the highest rate of aberrance, followed by PTEN. One case of EBT exhibited β-catenin aberrance; this case also harbored morules, whereas no other EBT had morules or aberrant β-catenin (Fig. 1C). Marker aberrance was most frequent in endometrioid adenocarcinomas of the ovary, with PAX2 aberrance in 93.1% of cases, followed by PTEN (27.6%) and β-catenin (48.3%). At least 1 of the 3 markers was aberrant in 96.6% of adenocarcinomas (Fig. 1D); the one case with no marker aberrancy was FIGO 1. The 2/29 PAX2 nonaberrant adenocarcinomas (PAX2 expressors) had been clinically tested for mismatch repair (MMR) deficiency by IHC; both retained expression of all 4 MMR markers. Scoring for each case within the diagnostic categories is shown in a case matrix (Fig. 1E).

**FIGURE 2.**
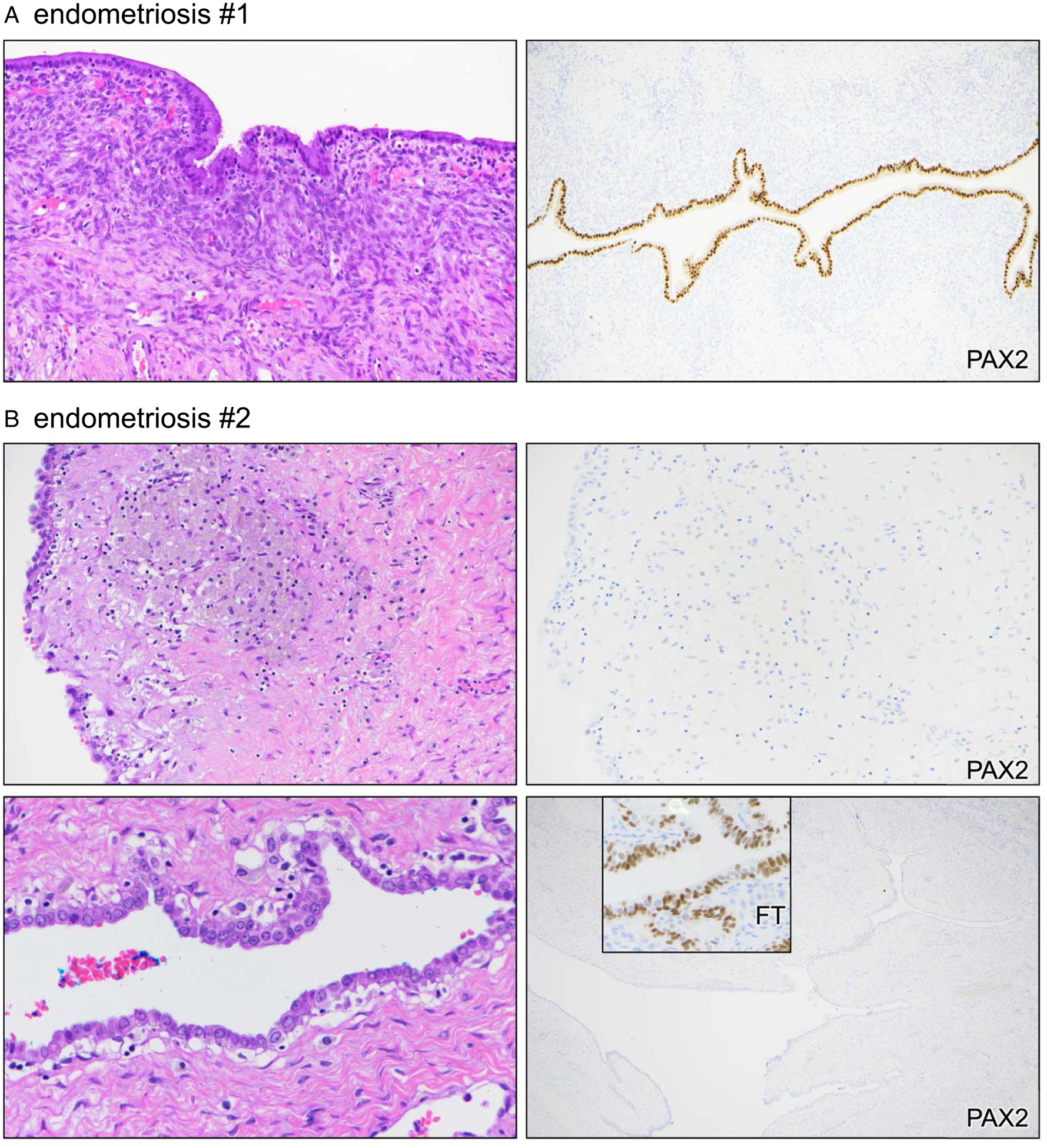
Marker aberrancy in nonatypical endometriosis. Two different cases are shown. (A) Case #1. (B) Case #2. Inset = fallopian tube (FT) internal control.

Whereas most cases of endometriosis strongly expressed PAX2 and were thus nonaberrant, the one case aberrant for PAX2 exhibited broad loss of PAX2 expression across the entire epithelial lining of the endometriotic cyst (100%, 2 cm cyst). PAX2 is normally expressed in secretory tubal epithelial cells, and a portion of fallopian tube on the slide served as an incidental positive control for PAX2 expression, confirming true loss of expression (Fig. 2A, B).

**FIGURE 3.**
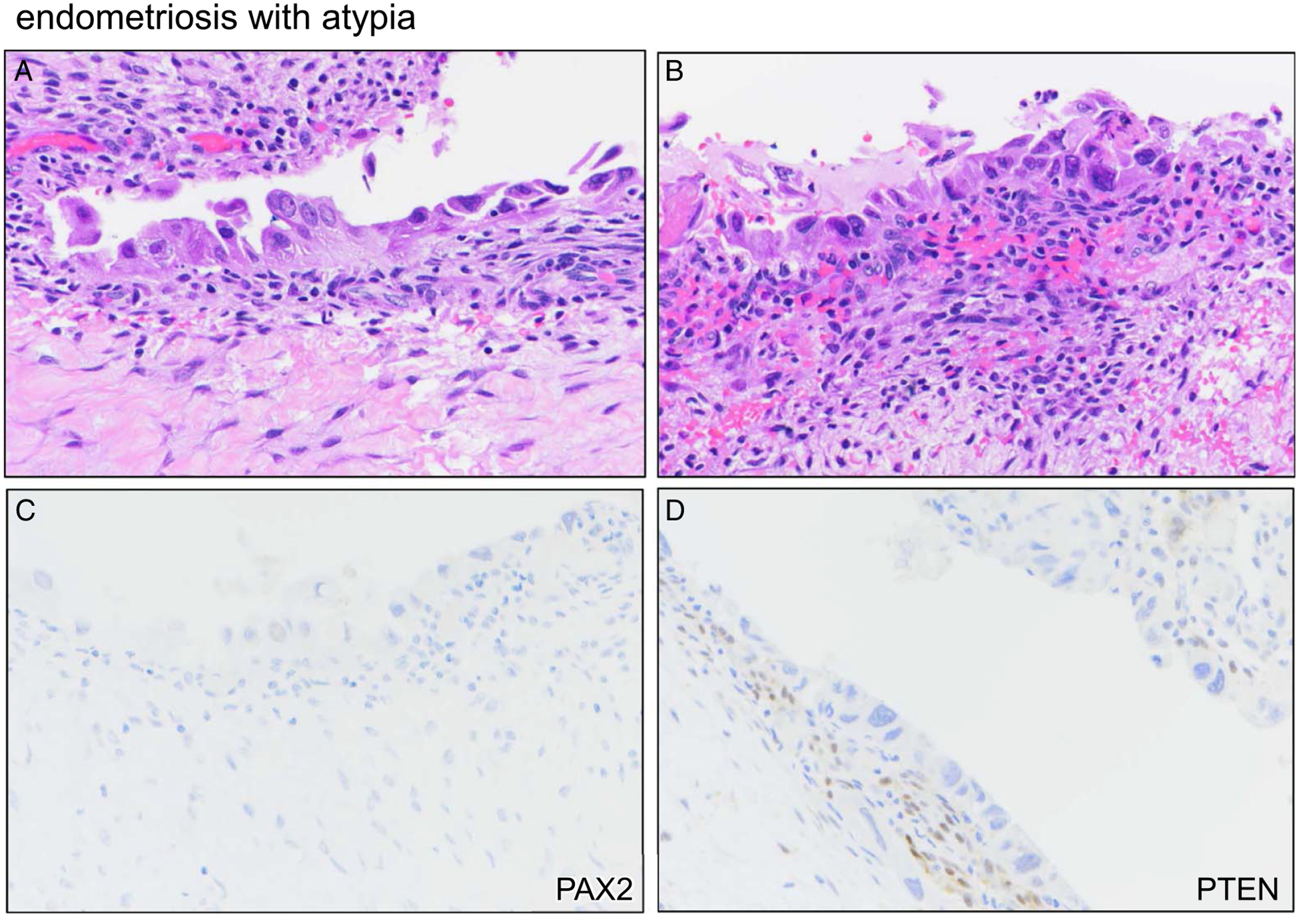
Marker aberrancy in a case of endometriosis with atypia. (A) H&E showing severe flat cytologic atypia. (B) H&E of different area from same case. (C). PAX2 immunostain, representative area. (D) PTEN immunostain, representative area.

Cases of EA were characterized by flat nuclear atypia with nuclear hyperchromasia and pleomorphism. Cells often appeared dyscohesive, with an apparent detachment of some cells (Fig. 3A, B). The single EA that exhibited marker aberrance resembled the other 4 cases with respect to histologic features and overall severity of nuclear atypia. This case exhibited nearly complete loss/aberrance (> 90% of epithelial cells) for both PAX2 and PTEN (Fig. 3C, D). The patient underwent hysterectomy 2 years later for failed medical management for endometriosis and persistent abnormal uterine bleeding; histopathologic findings included ovarian endometriosis with no evidence of malignancy.

**FIGURE 4.**
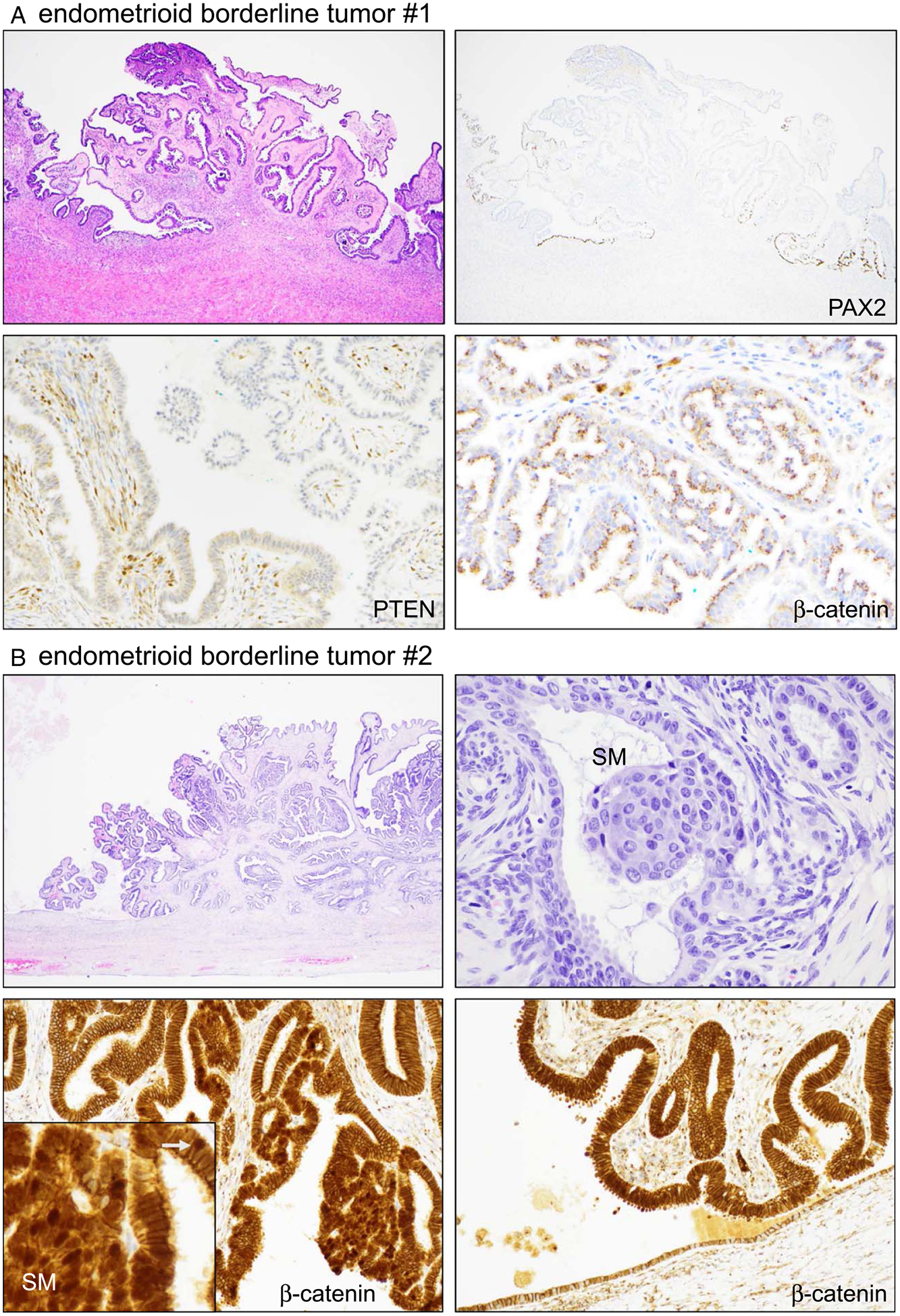
Marker aberrancy in the endometrioid borderline tumor. Two different cases are shown. (A) Case #1. (B) Case #2. higher magnification of SM; small white arrow points to nuclear localization of β-catenin in the adjacent epithelium. SM indicate squamous morule.

An example of EBT with seromucinous differentiation aberrant for PAX2 is shown in Fig. 4A. This case (#1), as well as all other EBT, was nonaberrant for β-catenin, exhibiting the normal (wild-type) pattern of cytoplasmic/membranous localization and no significant nuclear localization. PTEN expression was low, especially in cells with more abundant mucin, but there was definitive cytoplasmic staining, indicating non-aberrance (Fig. 4A). Only 1 EBT exhibited β-catenin aberrance, with distinct overexpression and nuclear localization in the nonmorular epithelium. This case also exhibited morules with nuclear β-catenin (Fig. 4B). Whereas in most cases patterns of marker aberrancy were preserved with the adjacent endometriosis (see next paragraph), in this case, the adjacent endometriotic epithelium was nonaberrant for β-catenin, suggesting that a *CTNNB1* mutation might have been the instigating molecular driver event within the endometriosis driving the formation of the EBT.

**FIGURE 5.**
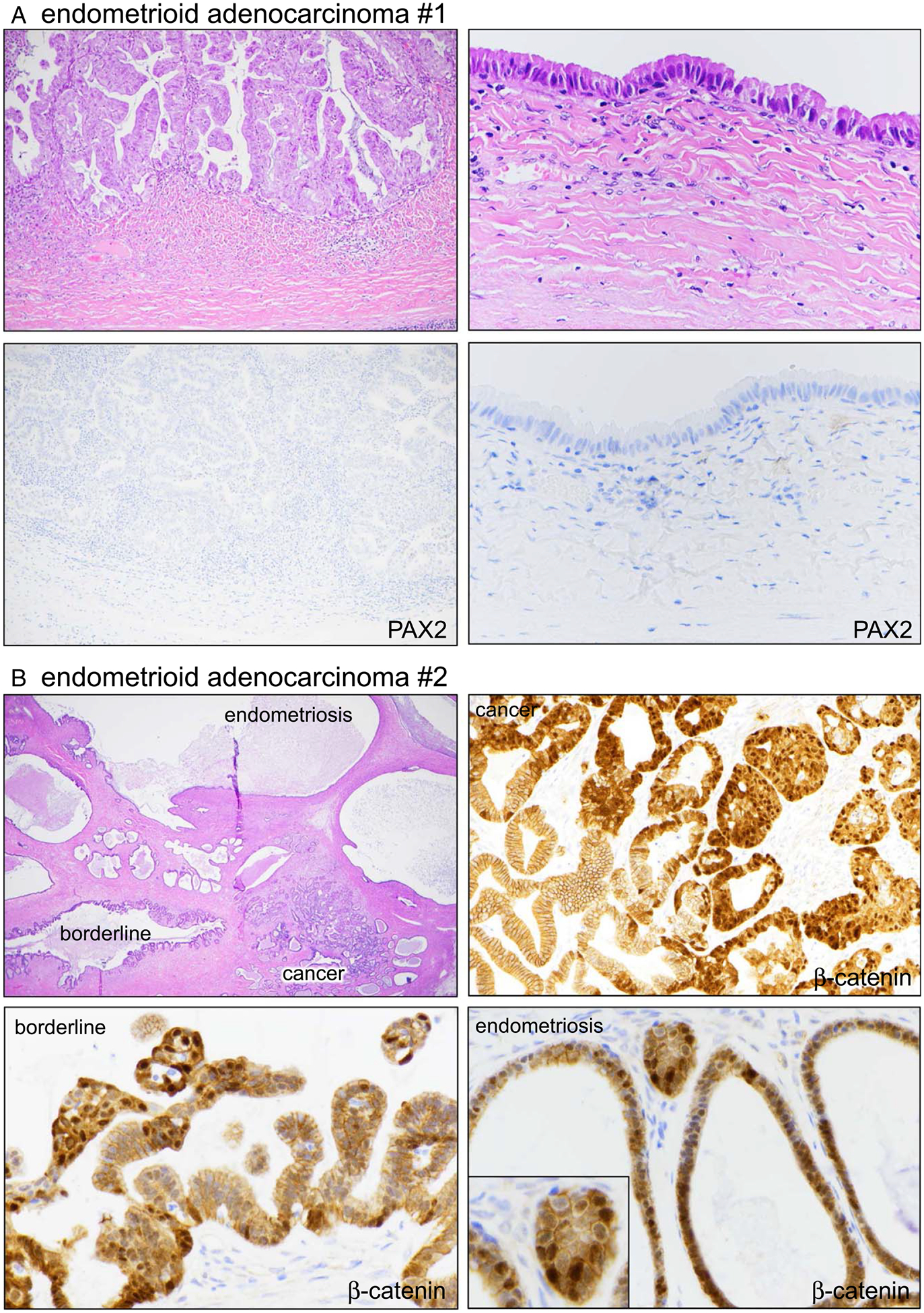
Marker aberrancy in 2 cases of endometrioid adenocarcinoma. (A) Case #1. Right-sided panels are of adjacent non-atypical endometriosis. The lower IHC panels correspond to the same areas shown in the H&E images. (B) Case #2. The single field in the H&E section shows distinct areas of adenocarcinoma, EBT, and endometriosis. β-catenin was aberrant across all areas.

In 6/19 EBT and 4/29 adenocarcinomas, distinct areas of adjacent endometriosis and/or EBT (in the adenocarcinomas) were identifiable. Patterns of marker aberrancy were preserved in these areas (with the case above being an exception), arguing that the definitive neoplasms arose from precursors where initiation was driven by the aberrancy of the relevant markers. For example, 1 case of PAX2-aberrant adenocarcinoma was associated with nonatypical endometriosis similarly characterized by PAX2 loss (Fig. 5A). In another adenocarcinoma that was characterized by squamous morules and β-catenin aberrancy, β-catenin was similarly aberrant in the normal-appearing adjacent endometriosis glands and the EBT areas (Fig. 5B). PAX2 was similarly aberrant across all components (benign, EBT, adenocarcinoma) (not shown) strongly arguing for an origin in this adjacent endometriosis. In 8/10 of the EBT or adenocarcinoma cases with adjacent endometriosis, patterns of marker aberrancy were preserved in at least 1 marker, while 7/10 had identical marker patterns, supporting a mechanism in which benign-appearing precursors can harbor molecular alterations of the relevant factors promoting development into more malignant lesions.

## DISCUSSION

Our study found that scoring criteria previously defined for the 3 markers in the endometrium are applicable to endometriosis and its neoplastic derivatives^12,18,23,25^. The overall results recall findings for the 3-marker panel in the endometrium, where the incidence of marker aberrance increases along the histologic spectrum of neoplastic progression (normal→disordered→nonatypical→EIN/AH→ adenocarcinoma)^12^. Although significant differences in aberrancy rates for individual markers in ovarian versus endometrial lesions are noted (see below), the aberrancy rate of 97% in endometrioid adenocarcinomas of the ovary is comparable to the reported 93% rate in EIN^18^. The aberrancy rate for PTEN was lower across the spectrum in ovarian versus endometrial lesions (e.g., 27.6% in ovarian endometrioid adenocarcinomas [this study] relative to 59.5% in endometrioid adenocarcinomas^28^). This finding is consistent with a lower reported incidence of *PTEN* mutations in ovarian endometrioid adenocarcinomas (17%) versus endometrial endometrioid adenocarcinomas (67%; *P* value of < 0.0001)^29^. The β-catenin aberrancy rate we found in EBT (1/19 cases) is lower than reported in 1 small retrospective study of selected cases (n = 8)^13^, a discrepancy that may be explained by different case selection criteria and a high proportion of seromucinous differentiation among our cases.

The molecular basis of PAX2 loss in endometrial neoplasia remains unknown^23^. Nonetheless, our findings suggest that PAX2 loss is also an important driver of ovarian endometrioid neoplasia. PAX2 loss occurred in 1 case of nonatypical endometriosis and 1 case of EA. The loss of PAX2 across the entire benign lesion suggests that PAX2 loss might be an early or instigating event in ovarian endometrioid carcinogenesis, as is well-established in eutopic endometrium. In the case of EA with PAX2 loss, the biological significance of PAX2 loss is substantiated by concurrent PTEN loss/aberrancy, providing further evidence that a subset of EA may be significant precursor lesions (ie, intraepithelial carcinomas). Most cases of EA likely represent reactive changes, but this case suggests that a subset represents true intraepithelial carcinomas, a question that warrants further investigation. While the 3x panel could be useful in identifying EA at higher risk of neoplastic progression, important caveats are that there is no consensus if or how EA should be reported by pathologists, or how it should be clinically managed, other than it should prompt additional tissue sampling to exclude occult malignancy^5^. EA may also serve as a precursor for clear cell adenocarcinoma, a distinct endometriosis-associated malignancy^4,5^, although we did not observe histologic features suggestive of clear cell neoplasia among our EA cases. The finding of similar patterns of marker aberrancy in endometriosis adjacent to EBT and adenocarcinoma, or EBT adjacent to adenocarcinoma, further cements a link between aberrancy for β-catenin, PAX2, PTEN, and cancer initiation/progression in ovarian endometriosis-associated neoplasia.

Significant limitations of our study are the small sample size and lack of systematic follow-up. Nonetheless, our findings provide support for the utility of the panel in endometriosis-associated neoplasms, such as difficult or ambiguous cases of EBT (analogous to EIN/AH), with the caveat that the sensitivity would be lower than for EIN/AH. In summary, our study adds to our understanding of the performance of the 3x biomarker panel in diverse diagnostic contexts.

## Figures and Tables

**TABLE 1.** Patient Demographics

	N =	Age range	Mean age
Endometriosis	32	18–65	37
Endometriosis with atypia	5	30–44	39
Endometrioid borderline tumor	19	21–66	44
Endometrioid adenocarcinoma	29	31–83	57

## References

[1] Pasalic E, Tambuwala MM, Hromic-Jahjefendic A. Endometriosis: Classification, pathophysiology, and treatment options. Pathol Res Pract 2023;251:154847. 37844487 10.1016/j.prp.2023.154847

[2] Bulun SE. Endometriosis. N Engl J Med 2009;360:268–279. 19144942 10.1056/NEJMra0804690

[3] Anglesio MS, Yong PJ. Endometriosis-associated ovarian cancers. Clin Obstet Gynecol 2017;60:711–727. 28990985 10.1097/GRF.0000000000000320

[4] Matias-Guiu X, Stewart CJR. Endometriosis-associated ovarian neoplasia. Pathology 2018;50:190–204. 29241974 10.1016/j.pathol.2017.10.006

[5] Ray LJ, Watkins JC. Atypical endometriosis: a review of an incompletely understood putative precursor of endometriosis-associated ovarian carcinoma. Diagnostic Histopathology 2023;29:450–457.

[6] Wright MF, Fitzlaff S, Wyeth A, Nuclear beta-catenin expression in endometrioid intraepithelial neoplasia (atypical hyperplasia) does not predict carcinoma on subsequent hysterectomy. Int J Gynecol Pathol 2021;40:240–247. 32897964 10.1097/PGP.0000000000000695

[7] McCluggage WG. Endometriosis-related pathology: a discussion of selected uncommon benign, premalignant and malignant lesions. Histopathology 2020;76:76–92. 31846535 10.1111/his.13970

[8] Wepy C, Nucci MR, Parra-Herran C. Atypical endometriosis: Comprehensive characterization of clinicopathologic, immunohistochemical, and molecular features. Int J Gynecol Pathol 2024;43:70–77. 37043650 10.1097/PGP.0000000000000952

[9] Nakamura K, Nakayama K, Ishikawa M, Genetic analysis and phosphoinositide 3-kinase/protein kinase B signaling pathway status in ovarian endometrioid borderline tumor samples. Oncol Lett 2018; 16:189–194. 29928400 10.3892/ol.2018.8626 PMC6006484

[10] Aguilar M, Zhang H, Zhang M, Serial genomic analysis of endometrium supports the existence of histologically indistinct endometrial cancer precursors. J Pathol 2021;254:20–30. 33506979 10.1002/path.5628 PMC8252414

[11] Mutter GL, Lin MC, Fitzgerald JT, Altered PTEN expression as a diagnostic marker for the earliest endometrial precancers. J Natl Cancer Inst 2000;92:924–930. 10841828 10.1093/jnci/92.11.924

[12] Aguilar M, Chen H, Sahoo SS, beta-catenin, PAX2, and PTEN panel identifies precancers among histologically subdiagnostic endometrial lesions. Am J Surg Pathol 2023;47:618–629. 36939046 10.1097/PAS.0000000000002034 PMC10101134

[13] Oliva E, Sarrio D, Brachtel EF, High frequency of beta-catenin mutations in borderline endometrioid tumours of the ovary. J Pathol 2006;208:708–713. 16429393 10.1002/path.1923

[14] Quick CM, Laury AR, Monte NM, Utility of PAX2 as a marker for diagnosis of endometrial intraepithelial neoplasia. Am J Clin Pathol 2012;138:678–684. 23086768 10.1309/AJCP8OMLT7KDWLMF

[15] Monte NM, Webster KA, Neuberg D, Joint loss of PAX2 and PTEN expression in endometrial precancers and cancer. Cancer Res 2010;70:6225–6232. 20631067 10.1158/0008-5472.CAN-10-0149 PMC2912978

[16] Nucci MR, Castrillon DH, Bai H, Biomarkers in diagnostic obstetric and gynecologic pathology: a review. Adv Anat Pathol 2003; 10:55–68. 12605088 10.1097/00125480-200303000-00001

[17] Strickland AL, Rivera G, Lucas E, PI3K pathway effectors pAKT and FOXO1 as novel markers of endometrioid intraepithelial neoplasia. Int J Gynecol Pathol 2019;38:503–513. 30256235 10.1097/PGP.0000000000000549

[18] Aguilar M, Chen H, Rivera-Colon G, Reliable identification of endometrial precancers through combined PAX2, β-Catenin, and PTEN immunohistochemistry. The American Journal of Surgical Pathology 2022;46:404–414. 34545858 10.1097/PAS.0000000000001810 PMC8860214

[19] Lucas E, Chen H, Sahoo SS, β-Catenin, PAX2 and PTEN panel in the diagnosis of endometrial precancers: a case-based review. Diagnostic Histopathology 2023;29:468–482.

[20] Costigan DC, Dong F, Nucci MR, Clinicopathologic and immunohistochemical correlates of CTNNB1 mutated endometrial endometrioid carcinoma. Int J Gynecol Pathol 2020;39:119–127. 30702464 10.1097/PGP.0000000000000583

[21] Kim G, Kurnit KC, Djordjevic B, Nuclear beta-catenin localization and mutation of the CTNNB1 gene: A context-dependent association. Mod Pathol 2018;31:1553–1559. 29795437 10.1038/s41379-018-0080-0 PMC6168348

[22] Cancer Genome Atlas Research N. Kandoth C, Schultz N, Cherniack AD, Integrated genomic characterization of endometrial carcinoma. Nature 2013;497:67–73. 23636398 10.1038/nature12113 PMC3704730

[23] Chen H, Strickland AL, Castrillon DH. Histopathologic diagnosis of endometrial precancers: Updates and future directions. Semin Diagn Pathol 2022;39:137–147. 34920905 10.1053/j.semdp.2021.12.001 PMC9035046

[24] Sahoo SS, Aguilar M, Xu Y, Endometrial polyps are non-neoplastic but harbor epithelial mutations in endometrial cancer drivers at low allelic frequencies. Mod Pathol 2022;35:1702–1712. 35798968 10.1038/s41379-022-01124-5 PMC9596374

[25] Lucas E, Niu S, Aguilar M, Utility of a PAX2, PTEN, and beta-catenin Panel in the diagnosis of atypical hyperplasia/endometrioid intraepithelial neoplasia in endometrial polyps. Am J Surg Pathol 2023;47:1019–1026. 37314146 10.1097/PAS.0000000000002076

[26] Kobel M, McCluggage WL, Minamiguchi S, Endometrioid borderline tumorWHO Classification of Tumours Editorial Board FGT. WHO Classification of Tumours Series, 5th edn. Lyon, France: International Agency for Research on Cancer; 2020: 56–57.

[27] Niu S, Lucas E, Molberg K, Morules but not squamous differentiation are a reliable indicator of CTNNB1 (beta-catenin) mutations in endometrial carcinoma and precancers. Am J Surg Pathol 2022;46:1447–1455. 35834400 10.1097/PAS.0000000000001934

[28] Wang L, Piskorz A, Bosse T, Immunohistochemistry and next-generation sequencing are complementary tests in identifying PTEN abnormality in endometrial carcinoma biopsies. Int J Gynecol Pathol 2022;41:12–19. 33720084 10.1097/PGP.0000000000000763

[29] McConechy MK, Ding J, Senz J, Ovarian and endometrial endometrioid carcinomas have distinct CTNNB1 and PTEN mutation profiles. Mod Pathol 2014;27:128–134. 23765252 10.1038/modpathol.2013.107 PMC3915240

